# Prior experience with target encounter affects attention allocation and prospective memory performance

**DOI:** 10.1186/s41235-022-00385-7

**Published:** 2022-05-07

**Authors:** Kara N. Moore, James Michael Lampinen, Eryn J. Adams, Blake L. Nesmith, Presley Burch

**Affiliations:** 1 Oklahoma State University, 116 Psychology Building, Stillwater, OK 74078 USA; 2 216 Memorial Hall, Department of Psychological Sciences, University of Arkansas, Fayetteville, AR 72701 USA; 3 210 McAlester Hall, Department of Psychological Sciences, University of Missouri, Columbia, MO USA

**Keywords:** Prospective memory, Experience, Prevalence, Expectations, Frequency, Meta-cognition

## Abstract

We examined how prior experience encountering targets affected attention allocation and event-based prospective memory. Participants performed four color match task blocks with a difficult, but specified prospective memory task (Experiment 1) or an easier, but unspecified prospective memory task (Experiment 2). Participants were instructed to search for targets on each block. Participants in the prior experience condition saw targets on each block, participants in the no prior experience condition only saw targets on the fourth block, and, in Experiment 2, participants in the mixed prior experience condition encountered some of the targets on the first three blocks, and saw all the targets on the fourth block. In Experiment 1, participants in the no prior experience condition were less accurate at recognizing targets and quicker to respond on ongoing task trials than participants in the prior experience condition. In Experiment 2, we replicated the effect of prior experience on target accuracy, but there was no effect on ongoing trial response time. The mixed experience condition did not vary from the other conditions on either dependent variable, but their target accuracy varied in accordance with their experience. These findings demonstrate that prospective memory performance is influenced by experience with related tasks, thus extending our understanding of the dynamic nature of search efforts across related prospective memory tasks. This research has implications for understanding prospective memory in applied settings where targets do not reliably occur such as baggage screenings and missing person searches.

## Significance statement

The research was motivated by the legal problem of missing targets in search tasks such as not noticing a missing person in one’s midst or illicit behavior or objects while performing another task. These targets rarely occur which may impact engagement and performance during future versions of the task. A prospective memory task involves a person forming an intention to complete a task (e.g., preventing a weapon from entering an event) and then engaging in other activities (e.g., helping to move the security line along) until the target appears, signaling the opportunity to complete the prospective memory task. The present research investigated how prior experience encountering targets affects attention to and performance on future tasks. Participants either encountered targets on four prospective memory blocks (prior experience), encountered some targets on the first three blocks and all targets on the final, critical block (mixed prior experience, Exp 2), or only encountered targets on the final, critical block (no prior experience). Prior experience encountering targets resulted in higher accuracy at recognizing targets than prior experience not encountering targets. In Experiment 1, which contained a difficult, but clear prospective memory task, participants in the prior experience condition devoted more attentional resources to searching for targets than participants in the no prior experience condition. This finding did not occur in Experiment 2, when the prospective memory task was easier, but less clear. Understanding how prior experience affects prospective memory performance can help us to work to improve performance at legally relevant prospective memory tasks.

## Introduction

Event-based prospective memory involves remembering to complete an intended action when the proper conditions occur (Einstein & McDaniel, 1990; Einstein et al., 1992; Ellis et al., 1999; Maylor, 1996, 1998; McDaniel et al., 1998). One critical feature of prospective memory is that the person does not exclusively pursue the fulfillment of the intended action until the proper conditions occur for them to engage in the intended action. Instead, they go about their day-to-day lives performing a variety of tasks until the conditions occur for the person to engage in their intended action thus fulfilling their intention.

In the laboratory, event-based prospective memory is commonly studied by providing participants with an ongoing task, such as a lexical decision task, to perform simultaneously alongside a prospective memory task, such as pressing a specific button when a designated target word appears in the ongoing task. In this paradigm, a participant would engage with the lexical decision task, judging letter strings as words or non-words, as the primary task while also being on the lookout for a designated target word (e.g., box). The participant is instructed to engage in the intended action, usually pressing a button on the keyboard, when they encounter the target word within the lexical decision task.

### Attention and event-based prospective memory

Researchers have found evidence that a person’s limited attentional resources are often recruited for both the ongoing task and successful prospective memory (Einstein & McDaniel, 2005; Smith & Bayen, 2004). Guynn’s (2003) two-process model operationalizes attentional resources as keeping the intention active in working memory while *monitoring* the environment for the target to perform the intended action. Performing prospective memory tasks alongside ongoing tasks results in “costs,” or slowing in participants’ response time on the ongoing task (Smith, 2003, see Anderson et al., 2019 for review). This phenomenon has been referred to as task interference and is posited to demonstrate that attentional resources are engaged in both tasks. There are circumstances where task interference is not present during successful prospective memory performance which has led to the interpretation that shared attentional resources are not necessary under these circumstances. These circumstances include when the target to fulfill the intended action is highly salient, when the target is highly related to the ongoing task, or when the ongoing task orients a person’s attention to the target (Einstein & McDaniel, 2005). When these conditions arise the person may spontaneously retrieve their intention without attentional resources. Spontaneous retrieval refers to the ability to recognize the target and engage in the intended action without using attentional resources to monitor for the target. More recent research has established that people can rely on both spontaneous retrieval and attention-based monitoring to complete a single prospective memory task (Scullin et al., 2013). While attention is often necessary for prospective memory, this does not guarantee that people will allocate the attentional resources necessary to result in successful completion of the prospective memory task.

### Metacognitive influences on event-based prospective memory

Metacognition, an individual’s thoughts about their thoughts, influences people’s decisions about attention allocation in prospective memory tasks, and this has been demonstrated primarily through task interference being affected by metacognitive manipulations (Rummel & Meiser, 2013). Koriat et al. (2004) posited that metacognitive judgments are influenced by experience and beliefs, or what they referred to as theory-based judgments. Existing research on the effects of various metacognitive influences on prospective memory can be organized into experience-based and belief-based influences. In the current research, we were interested in the influence of repeated failure to encounter targets, an extreme experience-based influence, on prospective memory.

### Belief-based metacognitive influences on prospective memory

In the domain of belief-based influences on prospective memory, researchers have focused on the influence of the expected demands of the prospective memory task and the expected context of the prospective memory task on prospective memory. Boywitt and Rummel (2012; Exp 1) manipulated the expected demands of the prospective memory task by influencing participants’ beliefs about their odds of encountering the targets words (10% vs. 90%). Anticipated demands based on the expected prevalence rate of the targets affected participants’ decision criteria. Rummel and Meiser (2013) found that participants’ beliefs about task demands affected attention allocation (or task interference).

Additionally, several studies have examined the effect of beliefs, via instating context expectations, about when the chance to perform the intended action will occur. Marsh et al. (2006) found that task interference was higher when a person was handling a stimulus that they associated with the prospective memory intention than when a person was handling a stimulus that they did not associate with the prospective memory intention (see also Lourenço et al., 2013; Lourenço & Maylor, 2014). Nowinski and Dismukes (2005) found that when the target and task were associated that prospective memory performance was better than when they were not associated. Meier et al., (2006; Exp 2) found that participants who knew the context that the target would occur in performed better on the prospective memory task and had higher task interference than participants who did not know the context that the target would occur. Ball et al. (2014) and Kominsky and Reese-Melancon (2017) manipulated context expectations and found that task interference was higher on the block that participants expected the targets to occur. In addition, Kominksy and Reese-Melancon (2017) found that context expectations influenced prospective memory performance. Therefore, context expectations have been found to influence prospective memory accuracy and task interference.

### Experience-based metacognitive influences on prospective memory

In the domain of experience-based influences on prospective memory, researchers have focused on when targets are presented relative to the onset of the tasks, how people respond to targets occurring after a prospective memory task has ended, and target frequency. This research has primarily focused on the effect of experience within a single prospective memory task. Researchers have found that participants adjust their attention allocation in response to their experiences within prospective memory tasks (Loft & Yeo, 2007; Loft et al., 2008; Scullin et al., 2013). The delay between the start of the ongoing task and the encounter of the first target impacts prospective memory performance (Conte & McBride, 2018; McBride et al., 2011). Research on commission errors, or whether people respond to targets that are displayed after the prospective memory task has ended, has found that the number of targets encountered in the task predicts the rate of commission errors. Commission errors are higher when fewer of the targets are presented in the prospective memory task (Bugg & Scullin, 2013; Streeper & Bugg, 2021).

Several studies have found participants adjust their attention allocation based on their experience with the frequency, or prevalence, of the prospective memory targets (Czernochowski et al., 2012; Horn & Bayen, 2015; Exp 2.; Loft & Yeo, 2007; Exp 3). Loft and Yeo (2007; Exp 3) found that target frequency (~ 1% vs. 3%) affected task interference and accuracy on the prospective memory task. Similarly, Horn and Bayen (Exp 2; 2015) found that higher target frequency (20% vs 3%) resulted in more task interference. In the visual search literature, this type of manipulation is referred to as a prevalence manipulation, and there is an abundance of support showing that low prevalence negatively impacts performance at detecting the targets (Biggs et al., 2014; Evans et al. (2013); Hout et al., 2015; Wolfe et al., 1998, 2005, 2007).

In addition, Scullin et al. (2013) found that attention allocation to a prospective memory task is dynamic across and within ongoing tasks. Participants completed multiple ongoing tasks while maintaining a prospective memory intention. Participants who spontaneously noticed the first prospective memory target initiated monitoring, as measured by task interference, indicating that the target prompted expectations of encountering additional targets and thus affected attention allocation. Importantly, this study profoundly demonstrates that attention allocation fluctuates on a single prospective memory task based on experience. Relatedly, Kulmann and Rummel (2014) found that a combination of belief-based metacognition, influenced by vague instructions provided at the beginning of the study, and experience-based metacognition impacted participants attention allocation within a prospective memory task.

Of most relevance to the current research, Loft and colleagues (2008) examined the effect of target presentation on task interference within a single task. In this study, half of participants encountered the prospective memory targets and half did not encounter the prospective memory targets. Participants who did not encounter prospective memory targets experienced less task interference on the ongoing task than participants who had encountered prospective memory targets. This study provides evidence that people may reduce the attention allocated to the prospective memory task when targets are not presented within a single task. We sought to extend this work by examining the question of how prior experience across multiple related prospective memory tasks affects attention allocation and performance on a future task.

Most research on prospective memory has focused on understanding prospective memory performance when the targets to perform a behavior are consistently and reliably presented to participants. In the current research, we were interested in examining how prospective memory performance is affected when targets are not reliably presented. In everyday life, targets may not appear as expected or at all. One highly consequential instance of this is prospective person memory, which is a specific type of prospective memory wherein a person intends to report a sighting of a person, such as in a missing or wanted persons case (Lampinen et al., 2009). In the case of searching for missing or wanted persons, a citizen may repeatedly form the intention to search, such as when one encounters posters at the grocery store, and yet never encounter the missing person. As with prospective memory research more generally, researchers have found that attention and monitoring play a substantial role in predicting sightings (Moore & Lampinen, 2019). In prospective person memory research, expectations of encountering the target person have been found to affect sighting rates (Lampinen & Moore, 2016; Moore et al., 2016, 2018). Along these lines, participants who had previously looked for but failed to find two target persons were less likely to sight a third target person than participants who had a chance to encounter the first person they had been asked to look for (Lampinen & Moore, 2016). We were interested in how participants would perform on a prospective memory task after completing highly related tasks wherein they did not have a chance to encounter all the targets, and whether attention allocation explains target accuracy in these circumstances. It is important to understand how repeated failure to encounter targets affects performance on later prospective memory tasks, because it will provide a basic foundation for understanding the odds of success at important prospective memory tasks after a person has had previous experience not encountering targets on related tasks. If repeated failure on highly related tasks reduces the dedication of necessary cognitive resources to a task, then authorities could strategize about how to address this problem in applied settings.

### The present research

When performing a prospective memory task, people rely on their experience to estimate the likelihood of encountering a target. In the current research, we examined whether participants would generalize from their experiences of failing to encounter all or some targets, by adjusting their attention allocation, on a final prospective memory task wherein the targets appeared. We examine this question in the context of a standard prospective memory paradigm.

In two experiments, participants completed four blocks of an attention-demanding prospective memory paradigm, searching for targets on each block. In Experiment 1, participants searched for a different set of specific and unrelated prospective memory targets on each block. In Experiment 2, participants searched for words belonging to two target categories on each block. We manipulated participants prior experience encountering targets in the first three blocks to measure how this impacted performance on the fourth, critical block. We manipulated prior experience by asking participants to look for targets that would or would not appear on the first three prospective memory blocks. On the fourth, critical block, all participants were asked to look for prospective memory targets that appeared during the block.

Based on previous work demonstrating an effect of expectations and experience on prospective memory performance and attention allocation, we hypothesized that prior experience would increase accuracy on and attention allocation toward the prospective memory task compared to no prior experience. Specifically, we expected prior experience to manifest in increased accuracy at identifying prospective memory targets (H1) and higher task interference (i.e., slower response time on the ongoing task) (H2) compared to no prior experience.

## Method

### Participants

To determine the sample size needed for statistical power (1 − *β*) of 0.9, we conducted an a priori power analysis based on the effect size of a similar study (Moore & Lampinen, unpublished data) *f* = 0.227, *α* = 0.05, and two between-subjects groups. The total sample size estimated for this power level with an effect of that size was 206 participants. Two hundred and five general psychology students participated for course credit. The sample was 70.2% (*n* = 144) female, and the average age was 19.85 years (*SE* = 0.15, Range = 18–35). The sample was Caucasian (74.6%), African American (5.4%), Hispanic (5.4%), Asian (5.4%), bi-racial (3.9%), mixed race (1.0%), American Indian (0.5%), or did not specify their race (1%). The majority of the sample was right-hand dominant (86.6%, *n* = 201) but a small minority of participants were left-hand dominant (11.2%) or ambidextrous (0.9%). The majority of the sample’s native language was English (88.3%). Participants who indicated that they did not speak English fluently (*n* = 1) or that they did not have normal color vision (*n* = 3) were excluded from analyses. Software errors led us to exclude eight participants. We excluded participants who scored less than 25% accurate on an ongoing task. The exclusion criteria were set before analyses were conducted. Therefore, a total of 183 participants’ data were submitted to our analyses, which still allowed us to detect a main effect of prior experience with a power of 0.86.

### Design

**Fig. 1:**
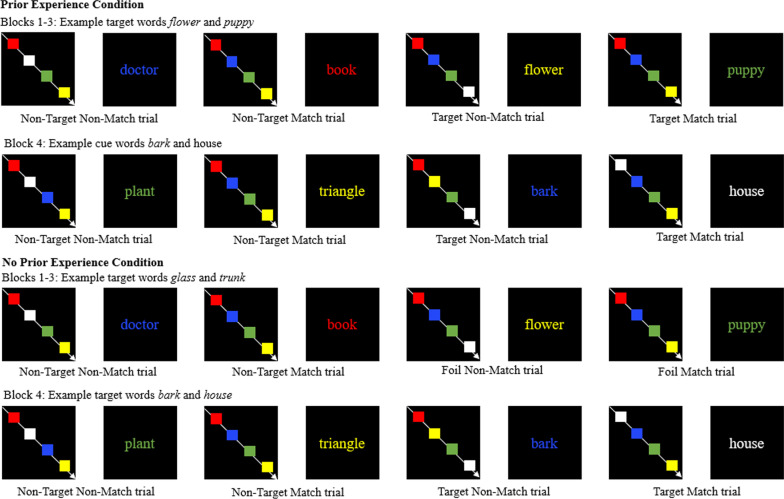
Example of trial types by block and condition in Experiment 1. *Note:* The colored rectangles each appeared by themselves on a screen followed by an interstimulus interval. They are presented all together here due to space constraints. The following comprises an example and may not correspond to words actually used on the task

Participants were randomly assigned to a prior experience or no prior experience condition. We manipulated prior experience in all conditions to exert experimental control over participants’ expectations. Participants completed ongoing color match tasks with embedded prospective memory tasks (Smith & Bayen, 2004). The color match task involves sequentially presenting participants with a series of four colored rectangles followed by a word printed in a colored font. The task is to indicate whether the word is printed in a color font that was displayed in one of the four preceding colored rectangle screens. The prospective memory task is to search for target words while performing the color match task. Participants completed four blocks of combined ongoing and prospective memory tasks. Participants were asked to search for a different set of target words that were semantically unrelated on each block. Participants in the prior experience condition had the opportunity to fulfill their prospective memory intentions on each block. Participants in the no prior experience condition, only had the opportunity to fulfill their prospective memory intentions on the fourth block because the prospective memory targets they were asked to be on the lookout for during the first three blocks never appeared (Fig. 1).

### Materials

Four blocks of color match tasks (Smith & Bayen, 2004) were designed using SuperLab. Two hundred and ninety-eight medium frequency words (*M* = 135.86) were randomly chosen (Kučera & Francis, 1967). Thirty-two words were used to create two color match practice blocks, 224 words were used in the color match tasks (56 in each block), and 42 words were used as prospective memory targets. The words in the four color match blocks were matched according to frequency and word length: block one (mean frequency = 134.61, mean word length = 6.44), block two (mean frequency = 135.97, mean word length = 6.21), block three (mean frequency = 134.56, mean word length = 6.11), and block four (mean frequency = 138.31, mean word length = 6.16). Six target words were used per block; plus, on the first three blocks, participants in the no experience condition were asked to look for separate sets of six target words that would never be presented. The target words were matched according to length and frequency. On the last block, both conditions were asked to look for the same prospective memory targets based on their counterbalance. The block order was counterbalanced and prospective memory target words were randomly assigned to a block. The prospective memory targets occurred on trials 10, 20, 30, 40, 50, and 60 of each block to maximize the distance between each prospective memory target word.

Each word was preceded by four color screens, each featuring one of five different colored rectangles (i.e., red, blue, green, yellow, and white) approximately 83 × 60 pixels (or 1.5in by 1.3in) in size, on a black background. The colors presented were randomly assigned. Each word was displayed in one of the five colors. Some words were displayed in the color of one of the four color screens that preceded it (i.e., color match), and some words were displayed in the one color that did not match any of the four color screens that preceded it (i.e., no match). Each word was randomly assigned to be a color match or no match. This led to four different trial types: match prospective memory targets, no match prospective memory targets, match words, and no match words.

Each color screen appeared for 500 ms followed by a black screen for 250 ms. Words appeared on the screen in 18 point font and remained on the screen until a response was made. A black screen appeared after the word screen for 1000 ms.

### Procedure

Experimenters obtained consent and demographic information from participants, and then participants began the experiment in SuperLab Version 4. Participants were instructed to press the ‘y’ key on the keyboard if the color of the word matched one of the four preceding color blocks and the ‘n’ key if the color of the word did not match one of the four preceding color blocks. Participants completed two color match tasks that consisted of six trials and 28 trials, respectively.

After the color match tasks, participants received instructions about the prospective memory tasks. Participants were asked to look for prospective memory target words while completing the color match task. All participants received the same instructions that established the expectation that one set of prospective memory targets would appear on each block. Participants were instructed to press the ‘~’ key if they spotted a prospective memory target word. Participants memorized the corresponding prospective memory target words to criterion and recalled them in order three times, to control for any effect of prior experience on retrospective memory, before beginning each block.

Before beginning the first block, participants had to correctly indicate the correct key to press if the word was (a) a match, (b) a non-match, or (c) a prospective memory target word. Participants were instructed to ask the experimenter if they did not know the answer. This ensured that all participants knew the correct key to press for each type of response on the tasks.

After completing each block, participants were asked to indicate the key they were asked to press if they saw a prospective memory target and to recall the prospective memory targets. Participants in the no prior experience condition only encountered prospective memory targets on the last block. Participants in the prior experience condition encountered prospective memory targets on all four blocks. After completing all four blocks, participants were asked if they felt suspicious while completing the study, if they encountered the prospective memory targets they were told to look for, and which task they thought was most important.

## Results

In this experiment, we examined how prior experience affected prospective memory performance and attention allocation on future prospective memory tasks. We hypothesized that prior experience would result in increased accuracy at identifying prospective memory targets (H1) and slower response time on the ongoing task (H2) in contrast to no prior experience.

The results are organized into four sections: prior experience and prospective memory performance (H1), prior experience and ongoing task performance (H2), prospective memory performance across tasks, and self-report responses. Prospective memory accuracy was calculated as the average accuracy for all target items in a block. Ongoing task accuracy was calculated as the average accuracy for all non-target items in a block. Response time was calculated as the average response time of accurate target (prospective memory) or non-target (ongoing task) responses in the block. We trimmed ongoing task response times that were three standard deviations beyond the average response time for the participant.

### Prior experience and prospective memory

We assessed the impact of prior experience of encountering targets on related blocks on future prospective memory performance by examining how participants performed on the final block, wherein all participants encountered target words. We conducted a 2 (target match vs. target no-match) × 2 (prior experience: experience or no prior experience) repeated measures ANOVA with prospective memory target accuracy on the final block as the dependent variable. As predicted (H1), participants in the no prior experience condition were substantially less accurate at identifying prospective memory target words (*M* = 0.24, *SE* = 0.03) than participants in the prior experience condition (*M* = 0.46, *SE* = 0.03), *F*(1, 181) = 24.18, *p* < 0.001, *ɳ*^2^_*p*_ = 0.118. The main effect of match, *F*(1, 181) = 3.06, *p* = 0.082, *ɳ*^2^_*p*_ = 0.017, and the interaction between match and experience conditions, *F*(1, 181) = 2.18, *p* = 0.141, *ɳ*^2^_*p*_ = 0.012, were not significant.

### Ongoing task performance

#### Accuracy

Next, we examined whether prior experience affected ongoing task performance across all blocks. Mean accuracy and response time rates by condition and block are displayed in Tables 1 and 2. A 2 (prior experience: yes, no) × 2 (color match: match, no match) × 4 (block) repeated measures ANOVA on ongoing task accuracy revealed a significant effect of match such that participants were more accurate on no match items (*M* = 0.92, *SE* = 0.01) than on match items (*M* = 0.84, *SE* = 0.01), *F*(1, 543) = 98.34, *p* < 0.001, *ɳ*^2^_*p*_ = 0.35. We found a significant effect of block, *F*(3, 543) = 23.07, *p* < 0.001, *ɳ*^2^_*p*_ = 0.113 (Greenhouse–Geisser).

**Table 1:** Ongoing task accuracy across blocks by experience condition in Experiment 1

	Block 1		Block 2		Block 3		Block 4	
	*M*	*SD*	*M*	*SD*	*M*	*SD*	*M*	*SD*
No prior experience								
Match	0.86	0.1	0.86	0.13	0.84	0.15	0.79	0.16
No match	0.93	0.08	0.93	0.1	0.91	0.1	0.89	0.11
Prior experience								
Match	0.84	0.1	0.87	0.1	0.87	0.1	0.83	0.13
No match	0.94	0.07	0.94	0.07	0.93	0.07	0.91	0.09

**Table 2:** Ongoing task response time across blocks by experience condition in Experiment 1

	Block 1		Block 2		Block 3		Block 4	
	*M*	*SD*	*M*	*SD*	*M*	*SD*	*M*	*SD*
No prior experience								
Match	1366.97	419.47	1199.58	413.53	1099.25	339.01	1150.54	398.44
No match	1368.29	405.16	1166.61	380.01	1089.53	396.92	1144.06	409.85
Prior experience								
Match	1497.56	468.43	1417.76	454.82	1396.47	433.8	1315.81	385.82
No match	1498.54	478.03	1477.37	534.53	1433.96	525.97	1354.35	446.78

Critically, we were interested in whether prior experience interacted with block to affect ongoing task accuracy on the final block, wherein all participants were shown prospective memory targets. We found a significant interaction between block and experience condition, *F*(3, 543) = 3.81, *p* = 0.014, *ɳ*^2^_*p*_ = 0.021 (Greenhouse–Geisser). Follow-up comparisons revealed that there was no effect of condition on the first block, *F*(1, 181) = 0.08, *p* = 0.784, *ɳ*^2^_*p*_ < 0.001, or the second block, *F*(1, 181) = 0.73, *p* = 0.396, *ɳ*^2^_*p*_ = 0.001. Participants in the no prior experience condition (*M* = 0.87, *SE* = 0.01) were less accurate on the third block than participants in the prior experience condition (*M* = 0.90, *SE* = 0.01), *F*(1, 181) = 4.19, *p* = 0.042, *ɳ*^2^_*p*_ = 0.023. Participants in the no prior experience condition (*M* = 0.84, *SE* = 0.01) performed similarly to participants in the prior experience condition (*M* = 0.87, *SE* = 0.01) on ongoing task accuracy on the final block, *F*(1, 181) = 3.84, *p* = 0.052, *ɳ*^2^_*p*_ = 0.021. There was no effect of experience condition, *F*(1, 181) = 2.18, *p* = 0.141, *ɳ*^2^_*p*_ = 0.012, no interaction between the match and experience condition, *F*(1, 181) = 0.002, *p* = 0.967, *ɳ*^2^_*p*_ < 0.001, match and block, *F*(3, 543) = 2.46, *p* = 0.066, *ɳ*^2^_*p*_ = 0.013, and no three way interaction between match, block, and experience condition, *F*(3, 543) = 2.18, *p* = 0.093, *ɳ*^2^_*p*_ = 0.012.

#### Response time

**Fig. 2:**
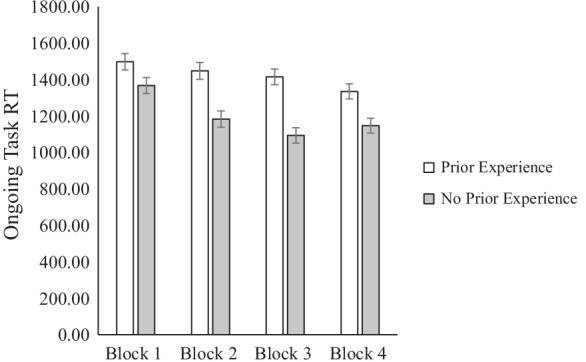
Ongoing task response time across blocks by experience condition in Experiment 1

A repeated measures ANOVA on ongoing task response time found an effect of block, *F*(3, 540) = 43.76, *p* < 0.001, *ɳ*^2^_*p*_ = 0.20 (Greenhouse–Geisser). There was also an effect of experience condition. Participants in the no prior experience condition (*M* = 1198.10, *SE* = 39.57) responded more quickly on the ongoing task than participants in the prior experience condition (*M* = 1423.98,  *SE*= 40.90), *F*(1, 180) = 15.75, *p* < 0.001, *ɳ*^2^_*p*_ = 0.080. Critically, we found a significant interaction between block and experience condition, *F*(3, 540) = 10.07, *p* < 0.001, *ɳ*^2^_*p*_ = 0.053 (see Fig. 2). Follow-up comparisons revealed that there was an effect of condition on the first block, *F*(1, 181) = 4.27, *p* = 0.04, *ɳ*^2^_*p*_ = 0.023, the second block, *F*(1, 181) = 17.51, *p* < 0.001, *ɳ*^2^_*p*_ = 0.088, the third block, *F*(1, 180) = 28.32, *p* < 0.001, *ɳ*^2^_*p*_ = 0.136, and the fourth block, *F*(1, 181) = 11.55, *p* = 0.001, *ɳ*^2^_*p*_ = 0.06. Participants in the prior experience condition responded more slowly to ongoing task trials than participants in the no prior experience condition and the size of this effect increased from the first block to the third block, but decreased in the fourth block. This finding is in line with hypothesis 2 that no prior experience encountering targets would result in less attention being allocated to the ongoing task than prior experience encountering targets.

Given that experience had an impact on response time even in block 1, we tested the impact of experience on response time before the first target was ever presented in block 1 to determine if the groups differed in performance before the introduction of the experience manipulation. We conducted a one-way ANOVA with experience as an independent variable on accurate ongoing task response time on the nine trials that appeared before the first target in block 1. We found no effect of experience on ongoing task response time before the first target in block 1, *F*(1, 181) = 0.03, *p* = 0.866, *ɳ*^2^_*p*_ < 0.001.

To further understand the interaction between block and prior experience, we examined the effect of block order by condition. In both conditions there was an effect of block. Participants in the prior experience condition showed a pattern of progressively speeding up, although only significantly so from blocks 3 to 4, *p* = 0.008. Whereas participants in the no prior experience condition progressively sped up on each block (block 1 *M* = 1567.63, *SE* = 39.89; block 2 *M* = 1183.09, *SE* = 39.33), except from the third block (*M* = 1094.39, *SE* = 39.85) to the fourth block (*M* = 1147.3, *SE* = 39.85), *p* = 0.155. This pattern of results indicates that participants in the no prior experience condition may have started monitoring during the final block, and thus did not continue the pattern of speeding up on the final block. There was no effect of match, *F*(1, 540) = 0.75, *p* = 0.388, *ɳ*^2^_*p*_ = 0.749, no interaction between match and experience condition, *F*(1, 540) = 3.23, *p* = 0.074, *ɳ*^2^_*p*_ = 0.018, no interaction between block and match, *F*(3, 540) = 0.18, *p* = 0.909, *ɳ*^2^_*p*_ = 0.001, and no three way interaction between match, block, and experience condition, *F*(3, 540) = 1.44, *p* = 0.231, *ɳ*^2^_*p*_ = 0.008.

To follow up on this pattern of findings, we analyzed the ongoing task response time data in bins by which target they preceded in the fourth block. This allowed us to examine the pattern of task interference across the fourth block. We conducted a 2 (experience: prior, no prior) × 6 (trial bin: trials preceding targets 1–6) mixed-effects ANOVA with trial bin as a within subjects variable. We found an effect of trial bin, *F*(5, 885) = 7.61, *p* < 0.001, *ɳ*^2^_*p*_ = 0.041, and condition, *F*(1, 177) = 11.12, *p* = 0.001, *ɳ*^2^_*p*_ = 0.059, but no interaction, *F*(5, 885) = 1.41, *p* = 0.217, *ɳ*^2^_*p*_ = 0.008. The two conditions followed a similar pattern of response time. Participants responded more slowly on the second bin of trials (*M* = 1369.23, *SE* = 50.14) which occurred after encountering the first target as compared to the first bin of trials before they encountered any targets (*M* = 1214.12, *SE* = 33.42), *p* = 0.002. This finding held when we tested the effect of target bin in the no prior experience condition alone (*p* = 0.015) and indicates that the no prior experience condition responded similarly to the first target as the prior experience condition, though the no prior experience was still monitoring less overall. In addition, participants responded more quickly on the first target bin (before encountering any targets) than in the second, third, or fourth target bins, *ps* = / < . 01. Participants sped up on the final target bin as compared to the second, third, or fourth target bins, *ps* < 0.01. Finally, and perhaps most interestingly, there was no effect of whether the participants in the no prior experience condition detected the first target on their response time to the second bin of trials, which occurred just after the first target was displayed, *F*(1, 92) = 0.16, *p* = 0.666, *ɳ*^2^_*p*_ = 0.002. Perhaps some participants in the no prior experience condition realized they had encountered the first target even if they missed it, and began engaging in monitoring for the remaining targets.

### Prospective memory performance across tasks

Next, we examined whether participants in the prior experience condition improved at the prospective memory task across blocks (see Table 3). A repeated measures ANOVA on prospective memory target accuracy revealed a significant effect of block, *F*(3, 261) = 5.19, *p* = 0.002, *ɳ*^2^_*p*_ = 0.056. Pairwise comparisons with Bonferroni corrections revealed that participants were more accurate on the first block (*M* = 0.55, *SE* = 0.03) than the third block (*M* = 0.46, *SE* = 0.03), *p* = 0.011, and the fourth block (*M* = 0.46, *SE* = 0.04), *p* = 0.024. These findings indicate that participants in the prior experience condition may have experienced a fatigue effect or may have experienced proactive interference across tasks. Participants were more accurate at responding to prospective memory target no match items (*M* = 0.53, *SE* = 0.03) than prospective memory target match items (*M* = 0.46, *SE* = 0.03), *F*(1, 261) = 11.44, *p* = 0.001, *ɳ*^2^_*p*_ = 0.116. There was no interaction between match and block, *F*(3, 261) = 0.799, *p* = 0.492, *ɳ*^2^_*p*_ = 0.009. Then, we examined response time to prospective memory targets across tasks in the prior experience condition (see Table 3). A repeated measures ANOVA revealed no effect of match, *F*(1, 114) < 0.001, *p* = 0.986, *ɳ*^2^_*p*_ < 0.001, no effect of block, *F*(3, 114) = 1.47, *p* = 0.239, *ɳ*^2^_*p*_ = 0.037 (Greenhouse–Geisser), and no interaction between match and block, *F*(3, 261) = 0.957, *p* = 0.388, *ɳ*^2^_*p*_ = 0.025 (Greenhouse–Geisser).

**Table 3:** Prospective memory accuracy and response time across blocks in the prior experience condition in Experiment 1

	Block 1		Block 2		Block 3		Block 4	
	*M*	*SD*	*M*	*SD*	*M*	*SD*	*M*	*SD*
Accuracy								
Match	0.51	0.33	0.5	0.36	0.42	0.35	0.42	0.36
No match	0.6	0.36	0.52	0.39	0.5	0.36	0.5	0.38
Response time								
Match	2309.67	1166.46	2325.06	1096.95	2220.13	925.2	2124.39	925.82
No match	2474.5	2048.21	2136.19	1193.79	2389.54	1540.38	1987.73	1097.56

### Self-report

At the end of the study session, we asked participants whether they thought the target words they were asked to look for were presented to them during the tasks (yes or no) and which task they thought was most important (ongoing task, prospective memory task, or both). We inadvertently failed to ask these questions to 23% of participants (*n* = 42) due to a software error. All but one these participants were in the prior experience condition. A chi-square revealed that participants in the prior experience condition were more likely to indicate that the target words were presented than participants in the no prior experience condition, *χ*^2^ (1, *N* = 139) = 6.27, *p* = 0.012, *Ф* = 0.212. A chi-square on task importance revealed no effect, *χ*^2^ (3, *N* = 140) = 6.96, *p* = 0.073, *Ф* = 0.22; 55% of participants in the no prior experience condition and 76% of participants in the prior experience condition prioritized the prospective memory task of searching for the target words.

## Discussion

The purpose of this research was to examine how prior experience with encountering prospective memory targets on separate but related tasks affected attention allocation and prospective memory performance on a future task. Participants completed four blocks of combined ongoing and prospective memory tasks. Participants in the no prior experience condition only had a chance to encounter prospective memory targets on the last block, allowing us to test the effects of repeated failure to encounter targets on related tasks. The effect of prior failure to encounter targets is important because it is analogous to the experience of repeatedly encountering missing and wanted persons alerts but not encountering the individuals themselves.

In support of hypothesis one, participants in the no prior experience condition were less accurate at noticing prospective memory targets on the final block than participants in the prior experience condition. In support of hypothesis two, we found that participants in the no prior experience condition responded more quickly to the ongoing task than participants in the prior experience condition, indicating that they devoted fewer attentional resources to completing the prospective memory task. The results from the ongoing task indicate that prior failure to encounter targets on highly related prospective memory tasks affected the attention participants allocated to a subsequent prospective memory task and thus their prospective memory performance. These findings demonstrate that prior experience with a prospective memory task may affect future prospective memory performance.

## Experiment 2

In Experiment 2, we maintained our original design and added a mixed experience condition to determine how performance is impacted when people have experience encountering one class of targets but no experience encountering another class of targets. All participants were asked to search for target words from two categories on each block. In the mixed experience condition, participants only encountered targets from one of the categories on the first three blocks.

We expected to replicate the findings from Experiment 1; prior experience would result in higher accuracy on the prospective memory task and slower response time on the ongoing task than the no prior experience condition. We expected that participants in the mixed experience condition would either perform similarly to the prior experience condition or would perform between participants in the no experience and prior experience conditions. If participants in the mixed experience condition learned that one category of targets was not being presented, then we expected that participants in the mixed experience condition would respond more quickly to the ongoing task than participants in the prior experience condition on the first three blocks, either because of perceived or actual reductions in prospective memory task demands. Monitoring for one set of targets may not be sufficient to account for noticing both sets of targets. If this is the case, then we would expect target accuracy to be lower in the mixed prior experience condition than in the prior experience condition on the final block. Wolfe et al. (2017) gave participants targets that appeared frequently and targets that appeared infrequently in a hybrid search task. They found that the targets that appeared infrequently were missed more than the targets that appeared frequently. Therefore, participants in the mixed prior experience condition may miss more of the targets from the no prior experience category than from the prior experience category on the last block. In addition, we would expect that the mixed prior experience condition would be more accurate at the prospective memory task and respond more slowly to the ongoing task than participants in the no prior experience condition. Alternatively, mixed prior experience participants may engage in the same amount of monitoring regardless of task demands or it may be the case that monitoring for one category of targets is sufficient to allow the noticing of the other category of targets. If either occur, then mixed prior experience participants should perform similarly to the prior experience participants on ongoing task response time and at detecting prospective memory targets on the last block.

## Method

### Participants

We estimated power based on the results of Experiment 1 for our primary analyses of interest on the effect of prior experience (3) on ongoing task response time and prospective memory target accuracy on the final, critical block. We used G*Power (Faul et al., 2007, 2009) to conduct an a prior power analysis for a one-way fixed effects ANOVA with prior experience (3) as a between subjects variable. We set power to 0.8, alpha to 0.05, and the effect size of *f* to 0.198. The estimated effect size was derived by converting the partial eta squared from Experiment 1 to Cohen’s *f* and then estimating the overall effect size as two-thirds of the effect in Experiment 1. We reasoned that the effect size should remain the same between the no prior experience and the prior experience conditions but the effect size difference between the mixed prior experience and the other two experience conditions would be half the size of the effect between the prior experience and the no prior experience conditions. The G*Power analysis resulted in an estimated sample size of 249 to power the analysis as 0.8 power.

Ultimately, 314 participants took part in the study, exceeding our sample size goal for 0.8 power. All participants were recruited from a university in the southern United States and took part in the experiment online due to the COVID-19 pandemic. Two participants reported issues with color perception, 37 participants did not complete the study in its entirety, and one participant completed the study a second time. Some of these participants also scored less than 25% accuracy on one of the ongoing task blocks. These participants data were excluded from the analyses. A total of 274 participants were included in the analyses.

### Design

**Fig. 3:**
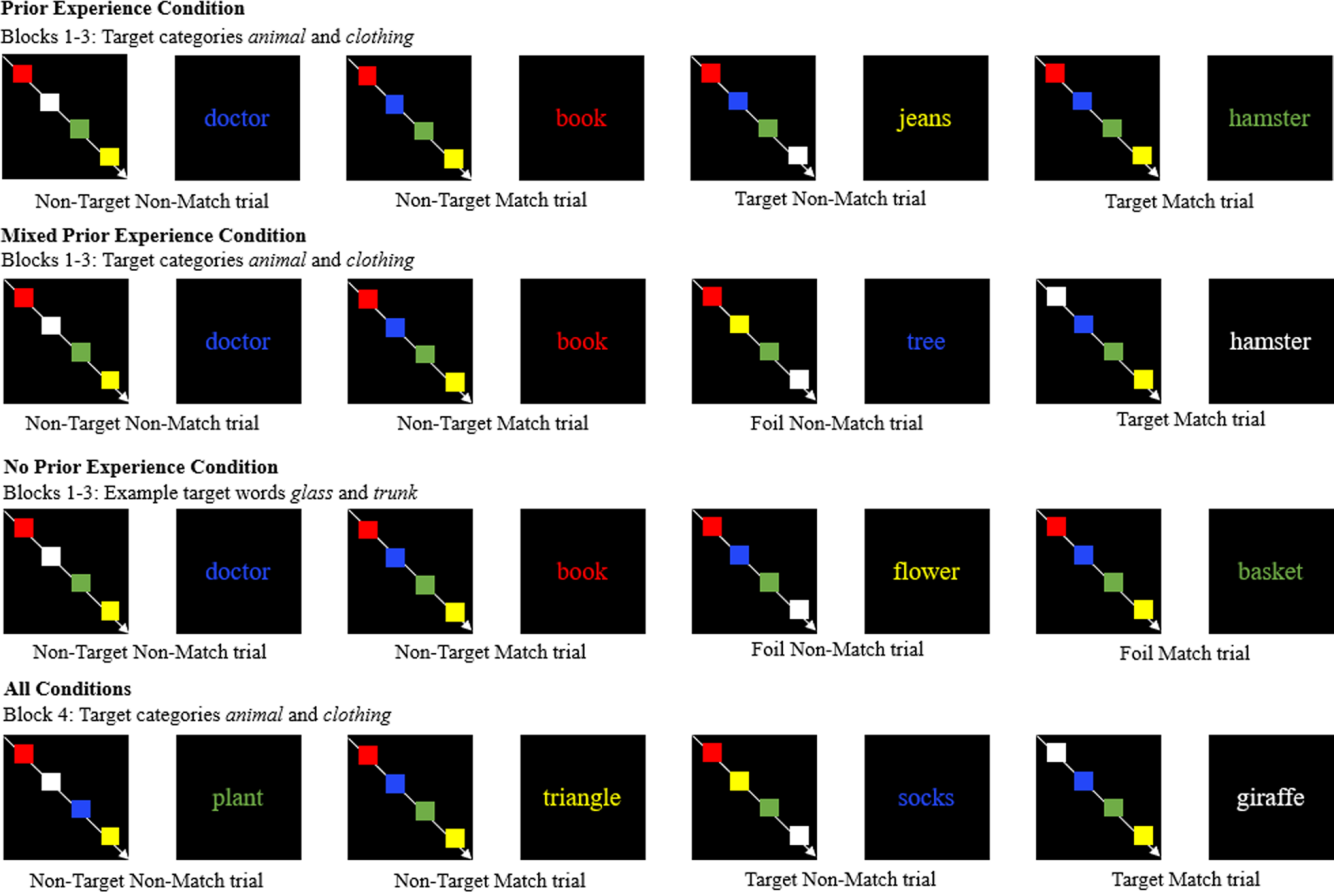
Example of trial types by block and condition in Experiment 2. *Note:* The colored rectangles each appeared by themselves on a screen followed by an interstimulus interval. They are presented all together here due to space constraints. The following comprises an example and may not correspond to words actually used on the task

The design of Experiment 2 was the same as the design for Experiment 1, with the addition of a mixed prior experience condition (Fig. 3). In Experiment 2, participants were asked to search for target words from the same two categories, *animals* and *clothing*, on each block. In the prior experience condition, on each block, six target words appeared, three from the category of *animal* and three from the category of *clothing*. In the mixed prior experience condition, three targets, all from only one of the categories, appeared on the first three blocks. The target category that the mixed prior experience participants encountered on the first three blocks was counterbalanced and randomly assigned. In the no prior experience condition, targets only appeared on the fourth block. In every condition, six targets, three from the category of *clothing* and three from the category of *animal*, appeared on the fourth block.

### Materials

Four blocks of color match tasks (Smith & Bayen, 2004) were designed using Millisecond’s Inquisit. Medium frequency words (*M* = 135.86) were randomly chosen (Kučera & Francis, 1967). Six target words were used per block, half from the *animal* category and half from the *clothing* category. The target word sets were matched according to length and frequency. In addition, a total of 18 words were used as fillers for the prospective memory targets on the first three blocks (6 on each block) in the no experience and mixed prior experience conditions. On the first three blocks, all six target words were replaced with non-target filler words in the no prior experience condition. On the first three blocks, there was a version that replaced all of the *animal* target words with non-target filler words and another version that replaced all of the *clothing* target words with non-target filler words in the mixed experience conditions.

There were 76 trials on each block: 70 non-target (35 match and 35 non-match) and six target (three from the animal category and three from the clothing category) or non-target filler trials. The prospective memory targets were presented in trials 16, 27, 38, 49, 60, and 71 in each block to maximize the distance between each prospective memory target word.

### Procedure

The procedure for Experiment 2 was the same as the procedure for Experiment 1 with the following exceptions. Participants were asked to look for the same two categories of prospective memory target words on each of the four color match blocks. All participants received the same instructions that established the expectation targets would appear on each block.

Participants were given example questions for each category (e.g., What key should you press if you see the word BIRD?, What key should press if you see the word SWEATSHIRT?) with feedback and validation. Then, participants completed a recall test for each category with feedback and validation. Once participants reported each category of words they were asked to look for they completed a block of trials. The recall test was given before the beginning of each block.

## Results

In this experiment, we examined how prior experience affected prospective memory performance and attention allocation on future prospective memory tasks. We hypothesized that prior experience would increase accuracy at identifying prospective memory targets (H1) and slow response time on the ongoing task (H2) relative to no prior experience. In addition, we hypothesized that the mixed prior experience condition would either perform similarly to the prior experience condition, or that the mixed prior experience condition would be less accurate at identifying targets and faster on the ongoing task than participants in the prior experience condition. We hypothesized that the mixed prior experience condition would be more accurate at identifying targets and slower on the ongoing task than participants in the no prior experience condition.

The results are organized into four sections: prior experience and prospective memory performance (H1), prior experience and ongoing task performance (H2), prospective memory performance across tasks, and self-report. The dependent variables were calculated using the same procedure as in Experiment 1.

### Prior experience and prospective memory

We assessed the impact of prior experience of encountering targets on related blocks on prospective memory performance by examining how participants performed on the final block, wherein all participants encountered target words. We conducted a 2 (target match vs. target no-match) × 3 (prior experience: prior experience, mixed prior experience, or no prior experience) repeated measures ANOVA with prospective memory target accuracy on the final block as the dependent variable. The main effect of prior experience was significant, *F*(2, 271) = 4.41, *p* = 0.013, *ɳ*^2^_*p*_ = 0.032. We followed up the significant main effect with Tukey’s post hoc tests. Replicating the finding from Experiment 1, participants in the no prior experience condition were less accurate at identifying prospective memory target words (*M* = 0.37, *SE* = 0.04) than participants in the prior experience condition (*M* = 0.52, *SE* = 0.04), *p* = 0.009. Descriptively, the mixed prior experience condition performed between the prior experience and no prior experience conditions, but there were no significant differences between mixed prior experience condition and the other experience conditions. The main effect of match, *F*(1, 271) = 2.04, *p* = 0.154, *ɳ*^2^_*p*_ = 0.007, and the interaction between match and experience conditions, *F*(2, 271) = 0.38, *p* = 0.686, *ɳ*^2^_*p*_ = 0.003, were not significant.

**Fig. 4:**
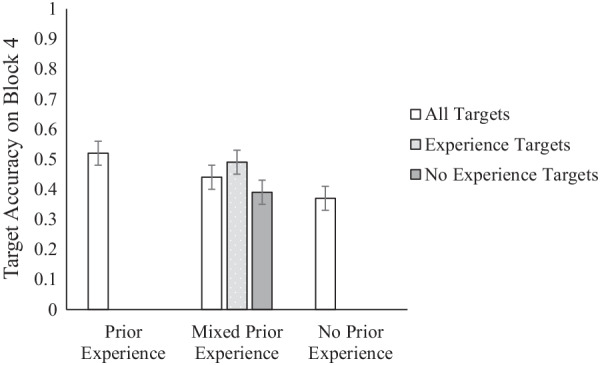
Target accuracy by experience condition and category in the mixed experience condition

To understand how the mixed prior experience condition participants performed on targets based on their prior experience with them, we conducted a one-way repeated measures ANOVA 2 (within condition experience: prior experience, no prior experience) on the mixed prior experience condition. We found that participants in the mixed prior experience condition were more accurate at detecting the prior experience targets (*M* = 0.49, *SE* = 0.04) than the no prior experience targets (*M* = 0.39, *SE* = 0.04), *F*(1, 89) = 6.79, *p* = 0.011, *ɳ*^2^_*p*_ = 0.071. In fact, the prior experience target accuracy (*M* = 0.49, *SE* = 0.04) in the mixed experience condition was similar to the target accuracy of participants in the prior experience condition (*M* = 0.52, *SE* = 0.04), and the no prior experience target accuracy (*M* = 0.39, *SE* = 0.04) in the mixed experience condition was similar to the target accuracy of participants in the no prior experience condition (*M* = 0.37, *SE* = 0.04) (Fig. 4).

### Ongoing task performance

#### Accuracy

Next, we examined whether prior experience affected ongoing task performance across blocks. Mean accuracy and response time rates by condition and block are displayed in Tables 4 and 5. A 3 (prior experience: yes, mixed, no) × 2 (color match: match, no match) × 4 (block) repeated measures ANOVA on ongoing task accuracy revealed a significant effect of block, *F*(3, 813) = 37.89, *p* < 0.001, *ɳ*^2^_*p*_ = 0.123 (Greenhouse–Geisser). Pairwise comparisons revealed a significant pattern of declining accuracy across blocks, all *ps* < 0.05, though the overall decline in performance was small from 87.4% accuracy on block 1 to 81.4% accuracy on block four. We found a significant effect of match. Participants were more accurate on no match items (*M* = 0.89, *SE *= 0.01) than on match items (*M* = 0.81, *SE* = 0.01), *F*(1, 813) = 127.13, *p* < 0.001, *ɳ*^2^_*p*_ = 0.008.

**Table 4:** Ongoing task accuracy across blocks by experience condition in Experiment 2

	Block 1		Block 2		Block 3		Block 4	
	*M*	*SD*	*M*	*SD*	*M*	*SD*	*M*	*SD*
No prior experience								
Match	0.84	0.12	0.83	0.14	0.82	0.17	0.75	0.19
No match	0.91	0.10	0.89	0.12	0.88	0.12	0.86	0.14
Mixed prior experience								
Match	0.82	0.13	0.81	0.17	0.79	0.18	0.74	0.22
No match	0.92	0.08	0.89	0.12	0.88	0.14	0.86	0.14
Prior experience								
Match	0.83	0.15	0.83	0.18	0.81	0.19	0.80	0.20
No match	0.92	0.08	0.90	0.13	0.89	0.12	0.87	0.14

**Table 5:** Ongoing task response time across blocks by experience condition in Experiment 2

	Block 1		Block 2		Block 3		Block 4	
	*M*	*SD*	*M*	*SD*	*M*	*SD*	*M*	*SD*
No prior experience								
Match	1308.50	424.38	1271.66	535.07	1210.78	610.98	1494.47	1535.80
No match	1233.44	387.68	1182.22	491.29	1291.24	1438.78	1754.42	4731.95
Mixed prior experience								
Match	1372.16	410.71	1471.10	1108.59	1463.93	991.05	1432.05	1089.21
No match	1400.69	640.22	1324.61	590.74	1486.10	1922.04	1507.34	1379.43
Prior experience								
Match	1437.15	419.59	1380.93	559.20	1321.50	444.90	1301.09	505.81
No match	1452.32	534.29	1380.05	692.51	1401.79	877.71	1378.97	735.79

Critically, we were interested in whether prior experience interacted with block to affect ongoing task accuracy on the final block, wherein all participants were shown prospective memory targets. The interaction between block and experience was not significant, *F*(6, 813) = 1.64, *p* = 0.148, *ɳ*^2^_*p*_ = 0.012 (Greenhouse–Geisser). There was no effect of experience condition, *F*(2, 271) = 0.56, *p* = 0.58, *ɳ*^2^_*p*_ = 0.004, no interaction between the match and experience condition, *F*(2, 813) = 1.03, *p* = 0.358, *ɳ*^2^_*p*_ = 0.008 (Greenhouse–Geisser), match and block, *F*(3, 813) = 3.78, *p* = 0.012, *ɳ*^2^_*p*_ = 0.014 (Greenhouse–Geisser), and no three-way interaction between match, block, and experience condition, *F*(6, 813) = 1.35, *p* = 0.238, *ɳ*^2^_*p*_ = 0.01 (Greenhouse–Geisser).

#### Response time

A repeated measures ANOVA on ongoing task response time revealed no effect of block, *F*(3, 810) = 1.21, *p* = 0.285, *ɳ*^2^_*p*_ = 0.004 (Greenhouse–Geisser). There was no effect of experience condition, *F*(2, 270) = 0.28, *p* = 0.76, *ɳ*^2^_*p*_ = 0.002. Descriptively, as in Experiment 1, the no prior experience condition slowed down from block 3 (*M* = 1251.01, *SE* = 102.89) to block 4 (*M* = 1624.45, *SE* = 200.45). There was no interaction between block and experience condition, *F*(6, 810) = 1.41, *p* = 0.242, *ɳ*^2^_*p*_ = 0.01. (Greenhouse–Geisser).

There was no effect of match, *F*(1, 810) = 0.373, *p* = 0.542, *ɳ*^2^_*p*_ = 0.001 (Greenhouse–Geisser), no interaction between match and experience, *F*(2, 810) = 0.13, *p* = 0.88, *ɳ*^2^_*p*_ = 0.001. (Greenhouse–Geisser), no interaction between block and match, *F*(3, 810) = 1.74, *p* = 0.183, *ɳ*^2^_*p*_ = 0.006 (Greenhouse–Geisser), and no three-way interaction between match, block, and experience, *F*(6, 810) = 0.40, *p* = 0.775, *ɳ*^2^_*p*_ = 0.003 (Greenhouse–Geisser).

### Prospective memory performance across tasks

Next, we examined whether participants in the prior experience condition improved at the prospective memory task across blocks (see Table 6). A repeated measures ANOVA on prospective memory target accuracy revealed no effect of block, *F*(3, 270) = 0.2.7, *p* = 0.052, *ɳ*^2^_*p*_ = 0.029. (Greenhouse–Geisser), no effect of match, *F*(1, 270) = 0.36, *p* = 0.552, *ɳ*^2^_*p*_ = 0.004, and no interaction between match and block, *F*(3, 270) = 0.52, *p* = 0.67, *ɳ*^2^_*p*_ = 0.006. (Greenhouse–Geisser).

**Table 6:** Prospective memory accuracy and response time across blocks in the prior experience in Experiment 2

	Block 1		Block 2		Block 3		Block 4	
	*M*	*SD*	*M*	*SD*	*M*	*SD*	*M*	*SD*
Accuracy								
Match	0.58	0.37	0.53	0.35	0.53	0.41	0.54	0.37
No match	0.49	0.38	0.51	0.37	0.49	0.38	0.49	0.40
Response time								
Match	3485.75	10645.54	1560.97	545.01	1577.98	556.40	1529.84	536.39
No match	1582.40	618.25	1712.23	782.21	1684.99	878.54	1548.39	485.14

Then, we examined response time to prospective memory targets across tasks in the prior experience condition (see Table 6). A repeated measures ANOVA revealed no effect of match, *F*(1, 99) = 1.08, *p* = 0.306, *ɳ*^2^_*p*_ = 0.032, no effect of block, *F*(3, 99) = 1.1, *p* = 0.302, *ɳ*^2^_*p*_ = 0.032 (Greenhouse–Geisser), and no interaction between match and block, *F*(3, 99) = 1.12, *p* = 0.299, *ɳ*^2^_*p*_ = 0.033 (Greenhouse–Geisser).

### Self-report

At the end of the study, we asked participants whether they thought the target words they were asked to look for were presented to them during the blocks of tasks (yes, no) and which task they thought was most important (ongoing task, prospective memory task, or both). A chi-square revealed that there were differences in reporting of the encountering of targets by experience condition, *χ*^2^ (2, *N* = 264) = 40.49, *p* < 0.001, *Ф* = 0.344; 81.39% of participants in the prior experience condition, 66.28% of participants in the mixed prior experience condition, and 41% of participants in the no prior experience condition reported that they thought all categories of targets were presented and did not note any exceptions. We conducted follow-up chi-square analyses including two prior experience conditions in each analysis, with Bonferroni corrections on our alpha level which led us to set our alpha level at 0.017. A chi-square revealed that participants in the prior experience condition were more likely to indicate that the target words were presented than participants in the no prior experience condition, *χ*^2^ (1, *N* = 178) = 29.94, *p* < 0.001, *Ф* = 0.41. A chi-square revealed that participants in the mixed prior experience condition were more likely to indicate that the target words were presented than participants in the no prior experience condition, *χ*^2^ (1, *N* = 178) = 11.14, *p* < 0.001, *Ф* = 0.25. A chi-square revealed that participants in the prior experience condition were no more likely to indicate that the target words were presented than participants in the mixed prior experience condition, *χ*^2^ (1, *N* = 172) = 5.09, *p* = 0.024, *Ф* = 0.172. A chi-square on task importance revealed no effect *χ*^2^ (6, *N* = 273) = 3.11, *p* = 0.795, *Ф* = 0.107; 51.11% of participants in the prior experience condition, 53.33% of participants in the mixed prior experience condition, and 49% of participants in the no prior experience condition thought searching for the target words was more important than the ongoing task.

## Discussion

The purpose of this experiment was to replicate the findings of Experiment 1 and to examine how mixed prior experience with encountering prospective memory targets on separate but related tasks affected ongoing task costs and prospective memory performance on a future task. As in Experiment 1, participants completed four blocks of prospective memory tasks. We replicated and extended the design of Experiment 1 by adding a mixed prior experience condition, in which participants had a chance to encounter one type of prospective memory target, but not the other on the first three blocks followed by a chance to encounter both types of targets on the last block. As in Experiment 1, we examined the effect of prior experience on prospective memory and ongoing task performance.

We partially replicated the results of Experiment 1. Participants in the prior experience condition were more accurate at detecting targets on the final, critical block than participants in the prior experience condition. The mixed prior experience condition did not differ in their accuracy from either of the other two experience conditions overall, but they did perform differently at recognizing the targets based on their experience with them and their performance on each category mapped onto the between subjects conditions by experience. There was no effect of experience on ongoing task response time. Perhaps the effect on response time was eliminated due to participants searching for categories of targets rather than multiple individual targets as in Experiment 1.

## General discussion

The purpose of this research was to examine how prior experience with encountering prospective memory targets on separate but related tasks affected attention allocation and prospective memory performance on a future task. Participants completed four blocks of prospective memory tasks. In Experiment 1, participants searched for specific target words; in Experiment 2, participants searched for words belonging to two target categories. Participants were asked to search for targets on four blocks of tasks. However, participants in the no prior experience condition only had a chance to encounter prospective memory targets on the last block (Experiments 1 & 2). Participants in the mixed prior experience condition encountered one category of targets on every block, but only had a chance to encounter the other category of targets on the last block (Experiment 2). Comparing the conditions to a prior experience condition allowed us to test the effects of repeated failure to encounter targets on related tasks. The effect of prior failure to encounter targets informs us about the effects of related experience on future prospective memory performance. It is analogous to the experience of repeatedly encountering missing and wanted persons alerts but not encountering the individuals themselves. Mixed prior experience encountering targets belonging to categories is analogous to the experience of searching for classes of targets with different prevalence rates, and is important when missing low prevalence classes of targets has high consequences, such as detecting suspicious behavior or problematic items in an airport scanner, while conducting an ongoing task.

In both experiments, we found support for the hypothesis that participants in the no prior experience condition were less accurate at noticing prospective memory targets on the final block than participants in the prior experience condition. In Experiment 1, participants in the no prior experience condition responded more quickly to the ongoing task than participants in the prior experience condition, indicating that they devoted fewer attentional resources to completing the prospective memory task. In fact, the effect was so strong that there were differences between the conditions on ongoing task response time starting in the first block, after the first target was displayed. The results from the ongoing task indicate that prior experience encountering targets on highly related prospective memory tasks affected the effort participants put into a subsequent prospective memory task. These findings suggest that prior experience with a prospective memory task may affect future prospective memory performance when attention benefits prospective memory. The latter finding was not replicated in Experiment 2 wherein participants searched for targets by category rather than individual instantiations of targets. Further, participants in the mixed experience condition did not perform differently from participants in the prior experience or no experience conditions in regard to overall prospective memory accuracy or ongoing task response time in Experiment 2. However, the mixed prior experience condition did perform similarly on target accuracy to the prior experience condition on targets they had prior experience encountering and to the no prior experience condition on targets they did not have prior experience encountering on the previous blocks. The prospective memory task was more difficult, but also more specified in Experiment 1 than Experiment 2, and we interpret the mixed results across experiments in light of those differences.

Capacity sharing theories like the preparatory attention model (PAM; Smith & Bayen, 2004), the multi-process model (Einstein & McDaniel, 2005), and the dynamic multi-process model (Scullin et al., 2013) of prospective memory specify that general attentional resources are sometimes or always necessary to complete prospective memory tasks. Evidence for capacity sharing comes from indirect measures of monitoring such as ongoing task response time in the presence of a prospective memory task and more direct measures such as eye tracking fixations onto prospective memory targets (Shelton & Christopher, 2016). Previous research has shown that people make decisions about how much attention to allocate to the prospective memory task (Kuhlmann & Rummel, 2014; Marsh et al., 2005; Rummel & Meiser, 2013). In addition, previous research has shown that experience and expectations about the prospective memory task impact attention allocation decisions and thus task interference and prospective memory performance (Boywitt & Rummel, 2012, Exp 1; Lourenço, et al., 2013; Kominsky & Reese-Melancon, 2017; Nowinski & Dismukes, 2005; Marsh et al., 2006; Meier et al., 2006, Exp 2; Rummel & Meiser, 2013). Kominsky and Reese-Melancon (2017) found that incorrect target expectations can negatively impact event-based prospective memory performance. Loft et al. (2008) demonstrated that not encountering targets reduces task interference on a single block. Our studies add to this body of work by demonstrating that when the tasks are difficult and well-specified that people will apply the knowledge gleaned from experience on recently completed, highly related tasks to a new task in the form of allowing that knowledge to influence decisions about attention allocation. The same effect was not observed when the tasks were easier and less well-specified, but an effect on prospective memory accuracy still occurred.

The dynamic multi-process model (Scullin et al., 2013) accounts for changes in monitoring based on experience within a prospective memory task. In the current study, we build on the dynamic multi-process model by demonstrating that participants changed their attention allocation, or monitoring, according to their past experiences across different, but highly related prospective memory tasks when the prospective memory task is difficult, but well-specified. In Experiment 1, in the no prior experience condition, response times on the ongoing task decreased, or sped up, on each block, except from the third block to the fourth, final critical block. This indicates that participants relied on spontaneous retrieval to an increasing degree, as their experience not encountering targets increased, until the fourth block. Participants had no way of knowing whether targets would appear on the fourth block so their slowing down indicates that some participants may have spontaneously noticed a target, inciting monitoring. Similarly, participants may have been engaged in some monitoring but increased their monitoring after encountering a target. In fact, participants did slow down after the first target was displayed in the final block. To our knowledge, this is the first research to examine the impact of repeatedly failing to encounter targets during prospective memory tasks on future related prospective memory task performance and attention allocation. We demonstrated that prior experience strongly influences performance on a future task by impacting attention allocation when the search task is difficult and well-specified.

Recently, an alternative account of task interference has been posited. Delay theory accounts posit that the cause of task interference is the time it takes to decide whether a stimulus is a target or not (Heathcote et al., 2015). The time it takes for evidence to accumulate about whether a stimulus is a target is posited to be longer than the time it takes to make an ongoing task decision. Strickland et al.’s (2018) prospective memory decision control (PMDC) theory expands on delay theory by incorporating Braver’s (2012) proactive and reactive control processes, which account for cognitive control in prospective memory and ongoing task decisions. Proactive control is an anticipatory process that directs attention to the prospective memory task (Strickland et al., 2018). It is similar to the concept of monitoring. Reactive control is a process that can inhibit accumulation of ongoing task decision information and excite accumulation of prospective memory decision information on prospective memory trials (Strickland et al., 2018). Accumulation models are needed to assess for these mechanisms and our studies (the first of which was conducted in 2015, the same year delay theory was proposed) were not set-up for accumulation modeling. However, we can consider how our findings fit with delay accounts. The findings from Experiment 1 can be explained from capacity sharing and delay accounts, as the same pattern of results is expected for either monitoring or proactive control. Participants in the prior experience condition were more accurate on the prospective memory task and experienced more task interference than participants in the no prior experience condition. This pattern of results can be explained by monitoring for the prospective memory targets or the adjustment of threshold to make a decision (i.e., delay theory accounts). Recent research by Boag et al. (2019) found evidence for both capacity sharing attention allocation mechanisms and delay theory response threshold mechanisms in prospective memory tasks. From the capacity sharing frameworks of the multi-process and dynamic multi-process theories, the lack of an effect of experience on ongoing task response time in Experiment 2 might be attributed to inadequate consideration of one’s experience or a general lack of monitoring. Similarly, from the delay theory framework, participants may not have taken their experience into account in determining their use of proactive control and decision thresholds. Our design cannot speak to reactive control processes; future research will need to include more trials to use accumulator models to measure these processes.

Surprisingly there was no effect of prior experience on attention allocation in Experiment 2. We propose several reasons why there was no effect of prior experience on task interference in Experiment 2 while accounting for the effect of prior experience on task interference in Experiment 1. Consider task differences between the experiments. In Experiment 1, participants searched for six unrelated targets on each block, and were told the exact targets they needed to search for before the block began. In Experiment 2, participants searched for two categories of targets to allow us to incorporate the mixed prior experience condition in the design. First, the categorical nature of the targets in Experiment 2 may explain the lack of effect. Marsh et al. (2003) found greater task interference on a prospective memory task where the target words were unrelated than one where they were related. Further, researchers have shown that targets differ in their monitoring difficulty (Scullin et al., 2010), and this can impact task interference. Therefore, in Experiment 2, participants may have relied more on spontaneous retrieval than monitoring, which could explain the lack effect of experience on task interference. Second, searching for targets that belonged to two categories may have been easier than searching for a different set of six unrelated targets on each block. Participants in Experiment 1 had to make seven decisions (i.e., ongoing task decision and six target decisions), whereas participants in Experiment 2 only had to make three decisions (ongoing task decision and two category decisions). The sheer number of decisions may have impacted the amount of monitoring one engaged in or their decision threshold. Third, the lack of specificity of the prospective memory task in Experiment 2 may have limited the impact of prior experience on monitoring: not knowing the exact targets to expect to encounter, how many targets to expect to encounter, or if one missed a target because the exact targets were not specified. Participants may not have been able to appropriately calibrate their monitoring by experience due to the lack of specificity of the prospective memory task. The number of targets one is assigned to search for impacts monitoring (Cohen et al., 2008) particularly when the targets are unrelated (Marsh et al., 2003, Experiment 2). The lack of effect of experience in Experiment 2 may be analogous to the lack of prevalence effects in face matching when no feedback is given (Bindemann et al., 2010; Papesh et al., 2018). Bindemann et al.’s conclusion was that the uncertainty of face matching limited the impact of prevalence on performance. Finally, Experiment 2 was conducted online whereas Experiment 1 was conducted in person. Completing the study online may have resulted in participants being less engaged in the study and therefore less impacted by the manipulations than if they had completed the study in the lab. In summary, in Experiment 2 we did not find evidence that participants calibrated their attention allocation based on their prior experience. These participants may have been relying on spontaneous retrieval or have been unable to properly calibrate their attention allocation because of the somewhat unspecified nature of the prospective memory task.

Despite the lack of effect of experience on monitoring in Experiment 2, prior experience impacted accuracy at detecting targets on the final block indicating that prior experience impacted some process or behavior important for prospective memory performance. In addition, when we examined performance at target accuracy in the mixed prior experience condition by experience, we found a difference in performance by experience. Specifically, we found that accuracy at the no prior experience targets was similar to the no prior experience condition’s target accuracy and that accuracy at the prior experience targets was similar to the prior experience condition’s target accuracy. This finding converges with Wolfe et al.’s (2017) finding in hybrid search tasks: participants missed more of the targets that appeared infrequently than the targets that appeared frequently. Given that there was no effect of experience on ongoing task response time, it is unlikely that attention allocation or speed-accuracy trade-offs account for the effect of experience on accuracy. Perhaps prior experience impacted participants ability to effectively engage in spontaneous retrieval. Research has shown that people can perform cognitive tasks more automatically (i.e., with fewer cognitive resources) with experience (Moors & De Houwer, 2006; Schneider & Shiffrin, 1977). The classic example of this being the STROOP interference effect, where people have difficulty inhibiting their automatic tendency to read printed color words so they may name the color font they are printed in (Stroop, 1935). Automaticity is not the only possible explanation though. Participants who had more experience may have memorized the target categories better than participants who experienced less success. Experience may also have impacted participants encoding strategy which could impact the chances that spontaneous retrieval was successful (Scullin et al., 2018).

Expectations and motivation are mechanisms that may have driven the effects of prior experience on prospective memory performance. There is a rich body of literature demonstrating that expectations influence attention allocation and performance on prospective memory tasks. These include studies of context expectations (Cook et al., 2005; Kominsky & Reese-Melancon, 2017; Meier et al., 2006; Nowinski & Dismukes, 2005), context associations (Lourenço et al., 2013; Marsh et al., 2006), expected demands (Boywitt & Rummel, 2012; Rummel & Meiser, 2013), and the frequency of target presentation (Loft & Yeo, 2007). The lack of effect of prior experience on task interference in Experiment 2, may be explained by participants not having enough information to properly calibrate their expectations. The overall effect of prior experience may have been limited because participants did not know how many targets to expect to encounter or which targets to expect to encounter. Relatedly, participants' motivation to put effort into the prospective memory task may have been impacted by the prior experience manipulation. It is less clear how the motivation account could explain the findings from both experiments though.

In addition to adding to theoretical knowledge of the impact of experience-based metacognition on prospective memory, we were also interested in the effects of prior failure to encounter targets because this is akin to legally applied search tasks. Specifically, Experiment 1’s design was analogous to the experience of citizens who encounter missing or wanted persons alerts. People repeatedly encounter alerts and yet recoveries via sightings occur infrequently. Although infrequent, recoveries via sightings occur, and field research has demonstrated that the majority of searchers will not notice a missing person in their midst even under very good conditions (Moore & Lampinen, 2019). As a result, it is important to understand what effect previous sighting failures may have on the odds of a sighting. Lampinen and Moore (2016) found that repeatedly encountering alerts without corresponding sightings reduced the likelihood of sighting a person they later encountered. The findings from Experiment 1 led us to conclude that repeated failure to encounter targets in difficult, but well-defined tasks leads people to reduce the attention they allocate to the prospective memory task in the future. Unfortunately, research in prospective person memory has demonstrated that the prospective memory task of searching for a missing person typically requires attentional resources (Moore & Lampinen, 2019). In addition, prior failure to encounter targets led to failing to notice targets when they were presented. This presents a problem for prospective memory tasks wherein the targets that one needs to search for may not often be presented, such as in missing and wanted persons searches.

The design of Experiment 2 was analogous to the experience of security and law enforcement officers being on the lookout for suspicious behavior or classes of unspecified objects as in monitoring crowds, live or recorded CCTV footage, and scanning baggage in screeners at the airport while conducting an ongoing task. Importantly, we know that encountering targets in these settings can occur at low prevalence rates and that this affects performance at detecting targets in visual and hybrid search tasks (Wolfe et al., 2005, 2017). The take away from the current study is that when these relatively unspecified search tasks are prospective memory tasks that accuracy, but not attention allocation to the prospective memory task may be impacted by prior experience on separate, but highly related tasks. While attention allocation was not impacted, accuracy was impacted by experience in this scenario, and this is problematic for highly consequential mistakes like failing to notice behavior that is a warning sign of someone being sex trafficked or failing to notice dangerous objects in baggage screenings. Even providing participants with mixed experience, encountering some classes of targets but not others, did not protect participants from the deleterious effects of prior experience not encountering a class of targets. Finally, we expect that when search tasks become more difficult like needing to be on the lookout for multiple classes of items, an effect on attention allocation may be seen as in Experiment 1, which may aggravate the effect of experience on target accuracy.

In the current research, we did not employ a traditional filler task before having participants begin the task. Many consider this to be a hallmark feature of the prospective memory paradigm. In the current research, a filler task presented multiple problems. The filler task could have had a greater impact on the first block than proceeding blocks. In addition, while filler tasks are designed to engage the mind to eliminate other content from working memory, it is difficult to ensure participants are truly engaged and participants in the prior experience block may have engaged with the filler task less than participants in the no prior experience block. When taken in the context of the larger psychology literature, the tasks we used still most closely resembles a prospective memory task and our results converge with related prospective memory studies. Additionally, people sometimes form intentions and then immediately begin a task wherein they have the chance to fulfill those intentions. Lastly, participants in our study performed comparably on all measures to other prospective memory studies. Most notably, participants in our study performed similarly, and in fact, somewhat worse, at allocating attention to the prospective memory task. This indicates that participants were not performing the tasks purely as a vigilance task.

## Conclusions

Overall, we found that a repeated lack of prior experience in encountering prospective memory targets when they were expected to appear reduced prospective memory accuracy, even within-subjects, on a final task wherein prospective memory targets appeared. In addition, when the prospective memory task was well defined participants who had prior experience encountering targets devoted more attention toward the prospective memory task than participants who had prior experiences wherein they did not encounter targets they were told to search for. This research has implications for understanding the impact of past experience on effort on related prospective memory tasks. This effect needs to be investigated further in the relevant applied contexts such as missing and wanted persons search and baggage screening to understand what impact failing to encounter targets has on future search performance and how to design interventions to reduce this problem.

## Data Availability

The datasets generated and/or analyzed during the current study are not publicly available due to the consent form not containing a disclosure that the data would be posted publicly.
